# Coordination between heart rate variability and physical activity may be diminished by fatigability in non‐older women in the hour before sleep

**DOI:** 10.14814/phy2.15126

**Published:** 2021-11-26

**Authors:** Kentaro Taniguchi, Akito Shimouchi, Naoya Jinno, Akitoshi Seiyama

**Affiliations:** ^1^ Human Health Sciences Graduate School of Medicine Kyoto University Kyoto City Japan; ^2^ College of Life and Health Science Chubu University Kasugai Japan; ^3^ National Cerebral and Cardiovascular Research Center Suita Japan; ^4^ Department of Bioscience Nagahama Institute of Bio‐Science and Technology Nagahama Japan

**Keywords:** cross‐correlation, fatigability, parasympathetic nervous system, physical acceleration

## Abstract

Fatigability is related to several diseases as well as the autonomic nervous system. We investigated whether fatigability is associated with coordination between physical acceleration (PA) and parasympathetic nervous activity (PSNA) in women. Overall, 95 women were divided into non‐old (*n* = 50; age: 22–59 years) and old (*n* = 45; age: ≥60 years) groups. PSNA and PA data were simultaneously obtained every minute for 24 h. We defined %lag0 as the percent ratio of lag = 0 min between PSNA and PA in 1 h. Cornell Medical Index was used to determine the degrees of physical and psychological symptoms. In the non‐older group in the hour before sleep, the participants with high fatigability scores had significantly lower %lag0 than those with low fatigability (*p* < 0.05). Additionally, those with higher fatigability combined with exhaustion in the morning had significantly lower %lag0 than those without exhaustion in the hour before sleep (*p* < 0.05) but not in the hour after waking up. These results suggest that fatigability in non‐older women was associated with loss of coordination between PSNA and PA in the hour before sleep. Additionally, exhaustion in the morning may be related to loss coordination of PSNA and PA during the previous night.

## Graphical abstract

We investigated whether fatigability is associated with the coordination between the physical acceleration (PA) and parasympathetic nervous activity (PSNA) in women. In the non‐older group in the hour before sleep, the participants with high fatigability scores had significantly lower coordination than those with low fatigability (*p* < 0.05). This result suggests that fatigability in non‐older women was associated with the loss of coordination between PSNA and PA in the hour before sleep.

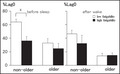

## 1 INTRODUCTION

Fatigue is one of the major symptoms in general conditions irrespective of health or illness. A large survey reported that half of the general population suffers from fatigue (Pawlikowska et al., 1994). There are some differences between fatigue and fatigability; therefore, assessment of fatigability can be used to better characterize fatigue (Murphy et al., 2016). Fatigability has been defined as the degree of fatigue experienced while performing a defined activity, which normalized fatigue to activity level (Eldadah, 2010). Therefore, understanding fatigability is vital in evaluating the impact of fatigue on physical activities and vice versa (Kim et al., 2018). Furthermore, it has been reported that the fatigue experienced in the morning may be distinct from that in the evening; however, both are closely related to each other (Dhruva et al., 2013).

Cornell Medical Index (CMI), a questionnaire devised for collecting a large amount of pertinent medical and psychiatric data with minimum expenditure of resources, includes a section on fatigability (Brodman et al., 1949). We used this questionnaire in this study as it has been widely used in clinical practice as a screening procedure to evaluate neurotic tendencies (Sawada et al., 2014). Furthermore, we employed the term “fatigability” based on its usage in CMI.

Fatigability in older people is different from that in younger ones (Santansato et al., 2015). There are age‐related differences in the neurovascular and neuromuscular systems between the two; therefore, older people exhibit different rates of fatigability than young adults during a fatigable task. However, the mechanisms underlying the age‐related differences in fatigability are not completely understood (Yoon et al., 2015).

Habitual physical activity and heart rate variability (HRV) have been reported to differ significantly between men and women (Aoyagi & Shephard, 2013; Koenig & Thayer, 2016). Broadly speaking, non‐older women are less fatigable than non‐older men during isometric fatiguing contractions (Hunter, 2014); therefore, fatigability also depends on the sex of the individual. Moreover, non‐older women with environmental factors, such as changes in hormones, could manifest alterations in their homeostasis. Furthermore, work‐related fatigue among non‐older women is complicated by the added work associated with housekeeping (Kivimäki et al., 2002). Therefore, evaluation of women's fatigability appears to require more objective analysis.

Several kinds of possible biomarkers and indexes for quantitative evaluation of fatigue have been reported (Japanese Society of Fatigue Science, 2011; Watanabe et al., 2008), such as, fatigue‐related oxidative stress, inflammatory markers, and autonomic nerve balance (Fukuda et al., 2016; Tanaka et al., 2011, 2015). Among them, the data on autonomic nerve balance based on electrocardiogram (ECG) are practically convenient.

HRV is a noninvasive tool to assess the variations in the beat‐to‐beat interval. It is used to assess cardiac autonomic functions and is related to the outcomes following cardiac events and stress (Klieger et al., 1992; Task Force of the European Society of Cardiology and the North American Society of Pacing and Electrophysiology, 1996). Most human studies on the HRV spectrum have examined the low‐frequency (LF: 0.04–0.15 Hz) and high‐frequency (HF: 0.15–0.40 Hz) spectral power bands (Task Force of the European Society of Cardiology and the North American Society of Pacing and Electrophysiology, 1996). A study has previously demonstrated that patients with chronic fatigue syndrome had significantly lower average daily physical acceleration (PA) (Sisto et al., 1998).

Cross‐correlation analysis gives a series of correlation coefficients between two‐time series by overlaying and temporally shifting the two series over a range of successive time lags (Chatfield, 1975). Cross‐correlation analysis is widely used to investigate the association in a physiological signal (Abdullah et al., 2010; Drinnan et al., 2001). Based on a cross‐correlation analysis between the time series of HRV and PA, coordination is defined as the time lag, and previously we found that the lag in older participants was significantly higher than that in the non‐older participants (Taniguchi et al., 2015). Therefore, we considered that the coordination between HRV and PA in daily lives is affected not only by aging, but also by mental or physical stress. We hypothesized that fatigability affects coordination between HRV and PA, that is, fatigability induces a mismatch between parasympathetic nervous activity (PSNA) and PA, thereby increasing the lag between them.

Previous studies have shown that immediately after the initiation of exercise, heart rate increases due to a withdrawal of PSNA and an increase in sympathetic nervous activity (Rowell & O'Leary 1990). PA causes an immediate change in HRV, whereas in impaired conditions, this response may be altered. Therefore, the lag may provide an index of coordination between PA and autonomic regulation of the cardiovascular system.

Thus, in the present study, we propose a new index, %lag0, which may indicate coordination between PA and PSNA. %lag0 was defined as the percentage of the lag = 0 min between PSNA and PA every 1 h. In this study, we investigated whether this method could be used to detect fatigability in non‐older women during free movement.

## 2 MATERIALS AND METHODS

### 2.1 Participants

**FIGURE 1:**
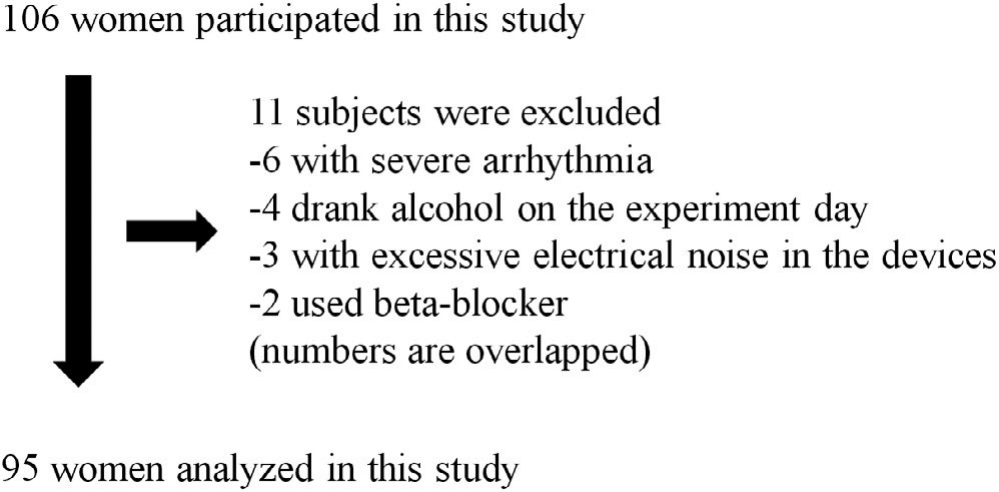
The study population for the evaluation of coordination between parasympathetic nervous activity and physical acceleration (PA)

Overall, 106 women (age: 20–85 years) volunteered to participate in this study. Based on medical interviews, physical examinations, laboratory blood investigations, and ECG, we excluded five participants who had consumed alcohol on the day of the experiment, six with severe arrhythmias, two who were on beta‐blockers, and three with excessive electrical noise in the devices described below (Figure 1). Arrhythmias were identified using a 12‐lead ECG. Finally, we examined 95 participants with the stated comorbidities. Of these, four had type‐II diabetes, 17 had hypertension, 35 had dyslipidemia, six had hepatic dysfunction, and two had renal dysfunction. However, these comorbidities were mild, and none of the subjects had restricted movements.

### 2.2 Protocols

The participants completed the questionnaire before or on the day of the assessments. They arrived at the laboratory at approximately 13:00 and underwent venous blood sampling and ECG. They wore a portable monitor (Active Tracer AC301; GMS Inc.) that recorded PA and R–R intervals over 24 h. During monitoring, the participants were instructed to continue their normal activities and avoid bathing. After the completion of the 24‐h monitoring, they returned to the laboratory. The experimental protocols have been described in detail previously (Taniguchi et al., 2015, 2021).

### 2.3 Questionnaires

We used the Japanese version of CMI (J‐CMI; Sankyobo Co. Ltd.), which was created by Kanehisa and Fukamachi (1972), to assess the physical and psychological symptoms (Brodman et al., 1949; Kanehira et al., 2001; Pendleton et al., 2004). J‐CMI for women comprises 213 questions in 18 sections. Fatigability included the following seven questions: “Q1. Do you often get spells of complete exhaustion or fatigue?”; “Q2. Does working tire you out completely?”; “Q3. Do you usually get up tired and exhausted in the morning?”; “Q4. Does every little effort wear you out?”; “Q5. Are you constantly too tired and exhausted even to eat?”; “Q6. Do you suffer from severe nervous exhaustion?”; and “Q7. Does nervous exhaustion run in your family?”. Each question invited “yes” or “no” as answers and was scored as one or zero, respectively. A yes response indicated that the individual has recently or previously experienced the symptoms (Brodman et al., 1949).

### 2.4 Physical acceleration

The body of the Active Tracer (GMS Inc.) equipped with a triaxial accelerometer (72 g) (Iwashita et al., 2003) was positioned on the frontal midline of the waist above the navel to avoid free movement of the device. The triaxial accelerometer obtained three‐dimensional accelerations every 40 ms with a sensitivity of 2 mG and a band‐pass filter of 0.3–100 Hz. The acceleration count was calculated as the average of the absolute values for acceleration in each direction for a given interval. Anteroposterior (*x*‐axis), mediolateral (*y*‐axis), vertical (*z*‐axis), and synthetic (synthesized triaxes as vector) accelerations were automatically calculated from the triaxial accelerometer. The data on the averaged synthetic accelerations during every 10 s were stored in the equipment. The absolute values of the resultant vector, which were calculated from the signals of triaxial acceleration, were averaged every 1 min (Tanaka et al., 2007).

### 2.5 Analysis

Spectral analysis of HRV was performed at 1‐min intervals using maximal entropy combined with the least square method (MemCalc System; Suwa Trust Co., Ltd.), and subsequently, separated into the HF and LF ranges for power analysis (Task Force of the European Society of Cardiology and the North American Society of Pacing and Electrophysiology, 1996). These indices were defined as HFnu = HF/(LF + HF). It is regarded as a marker of PSNA (Xhyheri et al., 2012). We estimated the times at which the participants fell asleep and woke up based on the changes in the body positions evaluated by the monitor.

### 2.6 Definition of %lag0

Lag was determined as the time difference indicated by the maximum correlation coefficient obtained from the cross‐correlation analysis between PSNA and PA (Chatfield, 1975). Cross‐correlation coefficients were calculated for 10‐min moving time windows over consecutive 60‐min periods. We defined impairments of coordination between PSNA and PA as a lag time of 1 min or longer. We used only the peak time lags that have reached statistical significance. The lag analysis has been discussed in detail in our previous study (Taniguchi et al., 2015). Subsequently, we defined %lag0 as the percentage of lag = 0 min in 1 h, and it is an index of coordination between PSNA and PA. Low levels of %lag0 indicated discoordination between PA and PSNA.

### 2.7 Statistical analysis

Data are expressed as mean ± standard error of the mean. Statistical analysis was performed by Mann–Whitney *U*‐test and Bonferroni procedure for multiple comparison correction using Excel and SPSS, appropriately. The significance of the cross‐correlation coefficient at each match position was evaluated based on Pearson correlation coefficients (Derrick et al., 1994). All *p* < 0.05 were considered statistically significant.

## 3 RESULTS

The participants were divided into the following four groups: group I, non‐older with low fatigability (age < 60 years and fatigability score < 3; *n* = 39); group II, non‐older with high fatigability (age < 60 years and fatigability score ≥ 3; *n* = 11); group III, older with low fatigability (age ≥ 60 years and fatigability score < 3; *n* = 31); and group IV, older with high fatigability (age ≥ 60 years and fatigability score ≥ 3; *n* = 14). There were no significant differences in age, body mass index, sleeping hours, and PA for 1 h (mG/h) both before sleep and after waking between groups I and II. In contrast, the older participants with high fatigability had significantly longer sleep duration and lower PA before sleep compared with those with low fatigability (Table 1).

**TABLE 1:** Comparison of the basic characteristics of age, body mass index, sleeping hours, and PA between fatigability scores

Group	I	II	III	IV
Fatigability score	<3	≥3	<3	≥3
Age (years)	<60	<60	≥60	≥60
Fatigability	<3	≥3	<3	≥3
*n*	39	11	31	14
Age	41.9 ± 1.9	44.9 ± 3.8	70.4 ± 1.0	70.7 ± 1.6
Body mass index	21.9 ± 0.5	22.4 ± 0.7	22.3 ± 0.5	23.9 ± 1.0
Sleeping hours (h)	6.9 ± 0.2	6.6 ± 0.6	7.2 ± 0.2	8.0 ± 0.3*
Frequency of nocturnal awakening	0.4 ± 0.1	0.8 ± 0.3	1.0 ± 0.1	1.2 ± 0.3
PA (mG/h)				
Before sleep	37.9 ± 2.2	42.9 ± 4.7	43.8 ± 2.0	33.2 ± 3.4*
After waking	51.3 ± 3.1	46.3 ± 4.4	54.8 ± 4.0	42.7 ± 4.7

Abbreviations:   PA, physical acceleration.

* *p* < 0.05 III versus IV.

**FIGURE 2:**
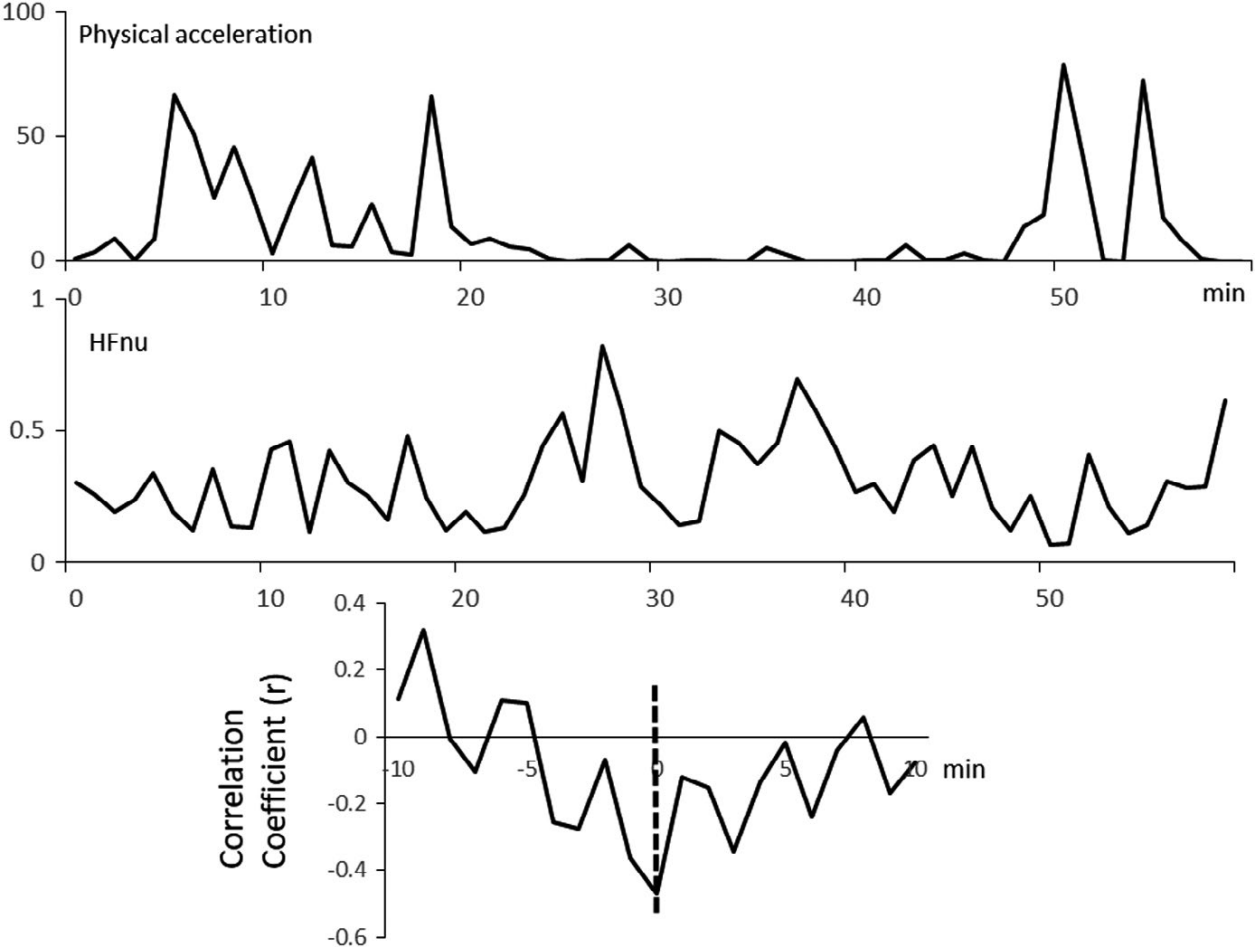
Lag = 0 in PA and HFnu before night sleep

**FIGURE 3:**
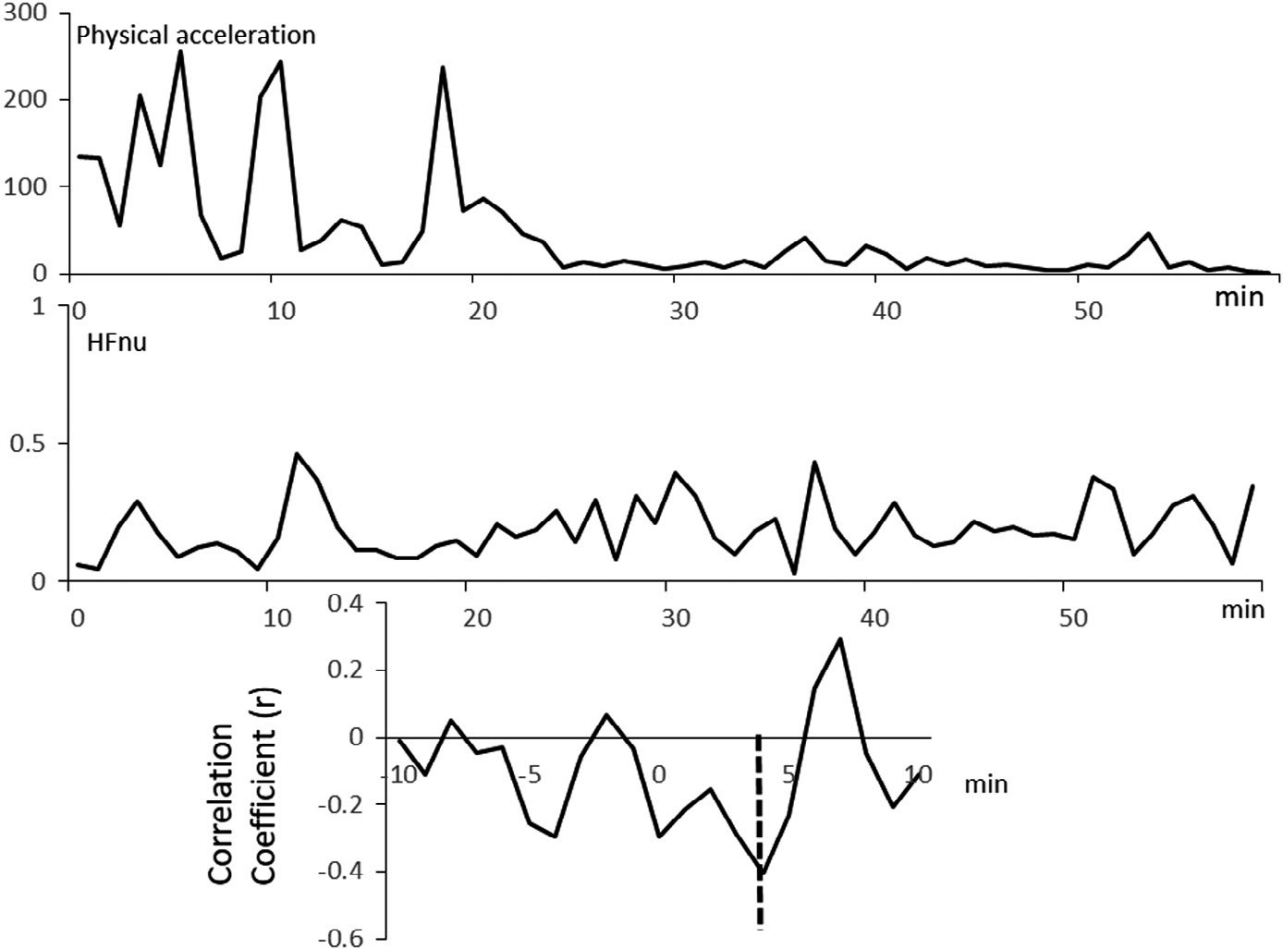
Lag = 4 min of PA and HFnu before night sleep

Low levels of %lag0 indicated discoordination between PA and PSNA. In a participant with low fatigability, the lag between the time series of PA and HFnu is shown in Figure 2. These indicate that the time series between PA and HFnu were coordinated, and the lag equals zero. If this coordination continues for 1 h, then %lag0 = 100 as defined in Section 2. Another example of a case with high fatigability with the time series and lag = 4 min of PA and HFnu is shown in Figure 3. These indicate that PA preceded in HFnu. In this case, %lag0 would be 0 if this lag continues for 1 h, and PA and HFnu would not be considerably coordinated. Detailed explanations are described in our previous paper (Taniguchi et al., 2015).

**FIGURE 4:**
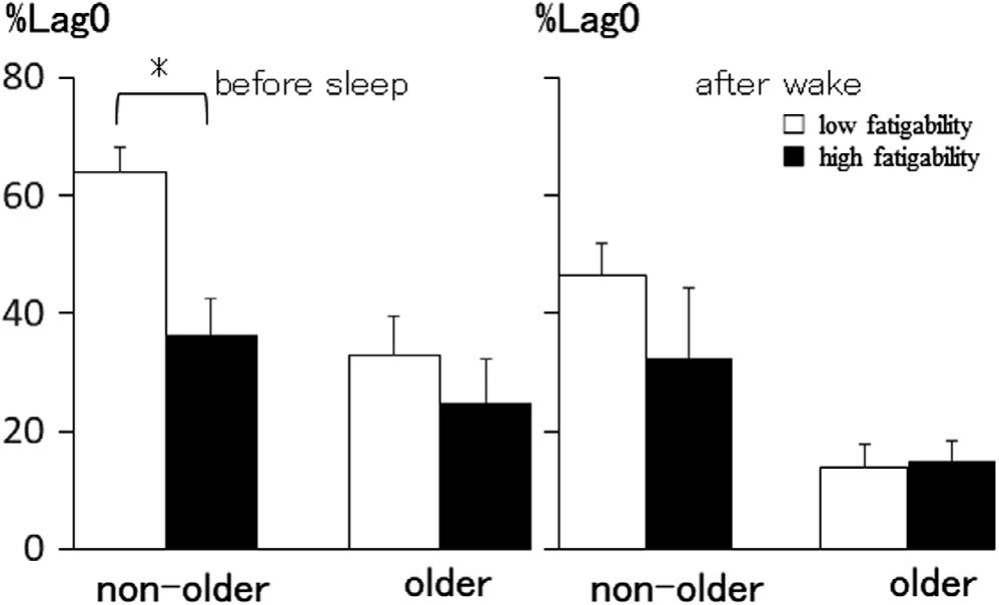
The relationship between %lag0 and fatigability during 1 h before and after sleeping in the non‐older and older groups. *p < 0.05 low fatigability versus high fatigability

In the hour before sleeping at night, non‐older participants with high fatigability had significantly lower %lag0 between HFnu and PA than those with low fatigability (left Panel in Figure 4). However, in the older groups, there were no significant differences in %lag0. In the hour after waking up, there were no significant differences in %lag0 between participants with low fatigability and those with high fatigability in the non‐older and older groups (right panel in Figure 4).

Of the 50 non‐older participants, the numbers of those who answered “yes” to the seven questions were 16, 16, 9, 10, 1, 2, and 4, respectively. Of the 45 older participants, 9, 20, 9, 13, 0, 1, and 3, respectively, answered yes to the same questions. Participants in the non‐older group who answered “yes” to Q2 (Does working tire you out completely?) had significantly lower %lag0 between HFnu and PA in the hour before sleep compared to those who answered “no”. However, in the older groups, there were no significant differences in %lag0 between PA and HFnu. Additionally, those in the non‐older group who answered “yes” for Q3 (Do you usually get up tired and exhausted in the morning?) had significantly lower %lag0 between HFnu and PA compared to those who answered “no”, whereas there were no significant differences in %lag0 before sleep in both questions 1 and 4, and Q1, Q2, Q3, and Q4 after waking up (Tables 2 and 3). Statistical analyses were not performed for the relationships between %lag0 and the answers to Q5, Q6, or Q7, because the numbers were small.

**TABLE 2:** %lag0 before sleep in response to the questionnaire items

Questionnaires	Non‐older		Older	
	No	Yes	No	Yes
Q1: Do you often get spells of complete exhaustion or fatigue?	63.0 ± 6.3	47.6 ± 13.9	31.0 ± 6.8	29.4 ± 8.5
Q2: Does working tire you out completely?	67.1 ± 6.2	38.8 ± 12.5*	32.4 ± 7.0	27.7 ± 8.4
Q3: Do you usually get up tired and exhausted in the morning?	66.2 ± 6.2	20.9 ± 10.1**	28.7 ± 6.3	36.7 ± 10.2
Q4: Does every little effort wear you out?	60.6 ± 6.4	47.7 ± 14.8	33.5 ± 6.9	22.4 ± 7.0

* *p* < 0.05

** *p* < 0.01, Answered no versus Answered yes.

**TABLE 3:** %lag0 after waking up in response to the questionnaire items

Questionnaires	Non‐older		Older	
	No	Yes	No	Yes
Q1: Do you often get spells of complete exhaustion or fatigue?	44.3 ± 5.4	40.8 ± 9.9	14.9 ± 7.2	30.0 ± 8.0
Q2: Does working tire you out completely?	44.1 ± 6.1	41.5 ± 9.9	14.4 ± 3.7	23.5 ± 7.5
Q3: Do you usually get up tired and exhausted in the morning?	45.3 ± 5.1	32.4 ± 12.6	16.1 ± 5.1	29.8 ± 13.5
Q4: Does every little effort wear you out?	45.7 ± 5.3	33.7 ± 12.9	24.6 ± 6.2	16.5 ± 7.9

## 4 DISCUSSION

The major finding of this study was that the participants in the non‐older group with high fatigability scores had significantly lower %lag0 compared with those with low fatigability in the hour before sleep; however, the difference was not significant in the hour after waking up. Additionally, participants in the non‐older group with higher fatigability and exhaustion in the morning had significantly lower %lag0 than those without exhaustion in the hour before sleep but not in the hour after waking up.

In our preliminary studies, we analyzed the lag between heart rate and PA. In non‐older group, an hour before sleep, Cohen's *d* effect size of %lag0 between heart rate and PA had a medium value. Almost all participants in non‐older and older groups indicated approximately %lag0 = 100 between PA and heart rate during 1 h before sleep and after waking up. Therefore, the frequency domain method was more specific than heart rate methods to detect the coordination.

In non‐older participants, no significant differences were observed in terms of basic characteristics between the high and low fatigability groups (Table 1). In the older group, however, the high fatigability group had significantly longer sleeping hours and lower PA in the evening than the low fatigability group. The sleeping hours at night also involved nocturnal awakening.

In the non‐older group, %lag0 1 h before sleep between HFnu and PA was significantly higher in the low fatigability group than that in the high fatigability group (Figure 4). There were no significant differences in the older group. It has been reported that fatigue stems not only from training overload or daily activities with inadequate rest, but also from various inputs, such as psychological stress (Meeusen et al., 2013). As a result, fatigability may be one of the factors that affect the coordination between HFnu and PA in non‐older women.

Participants who answered “yes” to the question 3 “Do you usually get up tired and exhausted in the morning?” had significantly lower %lag0 between HFnu and PA in the hour before sleep compared to those who answered “no”, but there was no significant difference in that during the hour after waking up (Tables 2 and 3). We considered that %lag0 in the hour before sleep and that in the hour after waking up had different clinical relevance since %lag0 of these two‐time periods had no significant correlation. Furthermore, in the hour after waking up, the participants generally performed a higher level of daily activities than they did in the hour before sleep (Table 1). Various social events occur during the daytime for most people, during which %lag0 varied markedly depending on the participants. Taking the above considerations together, our present results suggested that 1 h before night sleep appeared to involve less social activities and the most significant time for analysis of the relationship of fatigability and the coordination between HFnu and PA.

In this study, we focused on evaluating the relationship between fatigability and HFnu more clearly by using PA. It has been reported that altered autonomic nervous balance has been observed in adults with fatigue (Tanaka et al., 2011; Watanabe et al., 2008). Moreover, previous studies have shown that mental fatigability and altered HRV are both related to selective regions of the prefrontal cortex, which serves as a functional circuit (Leavitt & DeLuca, 2010). Additionally, another study showed that the medial orbitofrontal cortex is associated with fatigability in chronic fatigue syndrome (Tanaka et al., 2006). Therefore, we considered that fatigability may affect selective regions of the brain and/or neuromuscular system, and in turn this affects the coordination between HFnu and PA.

Our newly developed %lag0 index may be available in various kinds of clinical settings at least in non‐older women during free‐moving. Further studies are required to clarify the detailed physiological mechanism of discoordination between HRV and PA.

The analytical method employed in this study to evaluate fatigability and the coordination between autonomic activity and physical activity has limitations. This study did not include controls with matched comorbidities or physical conditions. Further controlled studies involving subjects with various metabolic and/or neuro‐muscular diseases that are associated with loss of coordination, are required.

## 5 CONCLUSIONS

Coordination between PSNA and PA diminished due to fatigability in non‐older women in the hour before sleep. Additionally, %lag0 can be estimated using a non‐invasive and simple method. This index could prove useful in public health or sports science disciplines to formulate measures to avoid fatigability.

## CONFLICT OF INTEREST

The authors declare that there is no conflict of interest.

## AUTHOR CONTRIBUTIONS

Kentaro Taniguchi conducted the procedures/methods of the study and prepared the manuscript. Akito Shimouchi developed the experimental plan and methodological procedures and revised the manuscript. Naoya Jinno contributed to the experiment and gave suggestions for presentation. Akitoshi Seiyama contributed to analysis and revised the original manuscript. All authors have read and approved the manuscript.

## PATIENT CONSENT STATEMENT

All participants provided written informed consent before participating in this study. All participants provided written and explicit consent for their anonymized data to be used in publications.

## Supporting information



Supplementary Material

## Data Availability

The data that support the findings of this study are available from the corresponding author, upon reasonable request.
