# Erratum

**DOI:** 10.14814/phy2.15196

**Published:** 2022-02-22

In the article by Taniguchi et al. (2021), the ethics approval statement was incorrectly omitted. The full statement is provided below.

## ETHICS APPROVAL STATEMENT

This study was approved by the Ethics Committee at the National Cerebral and Cardiovascular Research Center (M18‐19‐2, M26‐158), Chubu University (280031), and Kyoto University (R1758).

The publisher apologizes for the error.
