# Supplementary material for: Coordination between heart rate variability and physical activity may be diminished by fatigability in non‐older women in the hour before sleep
Source: Physiol Rep. 2021 Nov 26;9(22):e15126. doi: 10.14814/phy2.15126 (PMC8624186; doi:10.14814/phy2.15126)
Supplement: Supplementary file 1 — Supplementary Material [file PHY2-9-e15126-s001.pdf]

Appendix 1: Comparing %lag0 before sleep and after waking up

There were no significant differences between %lag0 after waking up in participants with high or low %lag0 before sleep in both the non-older and older groups. Therefore, we believe that %lag0 in the hour before sleep and after waking up may have different clinical meanings. We considered that the hour before sleep may be a recovery hour or a suitable hour during which there may be fewer confounding factors for smooth correlation between physical acceleration (PA) and heart rate variability (HRV). In contrast, smooth correlation during the initial hours after waking up may be easily affected by various internal and external environmental conditions.

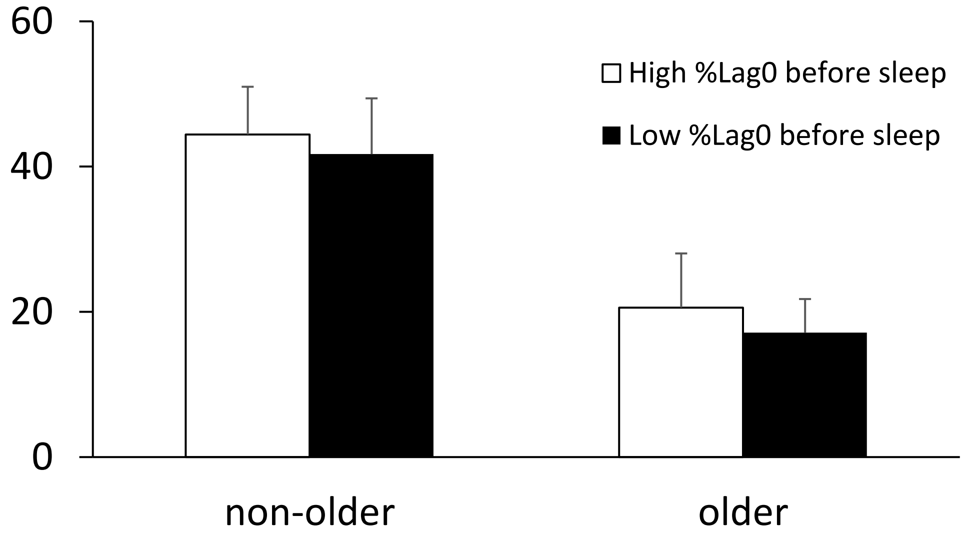

Supplemental Figure S1. %lag0 between HFnu and PA after waking up

Appendix 2 (only for reviewers): Relationship between sleep disorders and %lag0

Figure S2 depicts the relationship between %lag0 and nocturnal awakening. No significant difference was observed between the groups with (n = 17 in non-older group, 32 in older group) or without (n = 33 in non-older group, 13 in older group) nocturnal awakening.

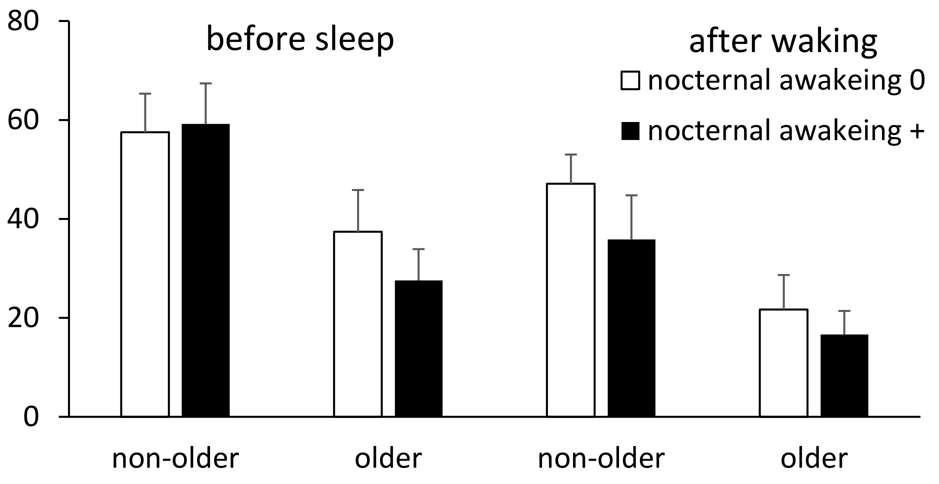

Supplemental Figure S2. %Lag0 and nocturnal awakening

Figure S3 depicts the relationships between %lag0 and sleep cycles based on our original questions “Is your sleep cycle is regular or irregular?”, although we could not obtain answers from all the participants. No significant differences were observed between the groups with regular (n = 26 in non-older group, 28 in older group) and irregular (n = 20 in non-older group, 14 in older group) sleep cycle.

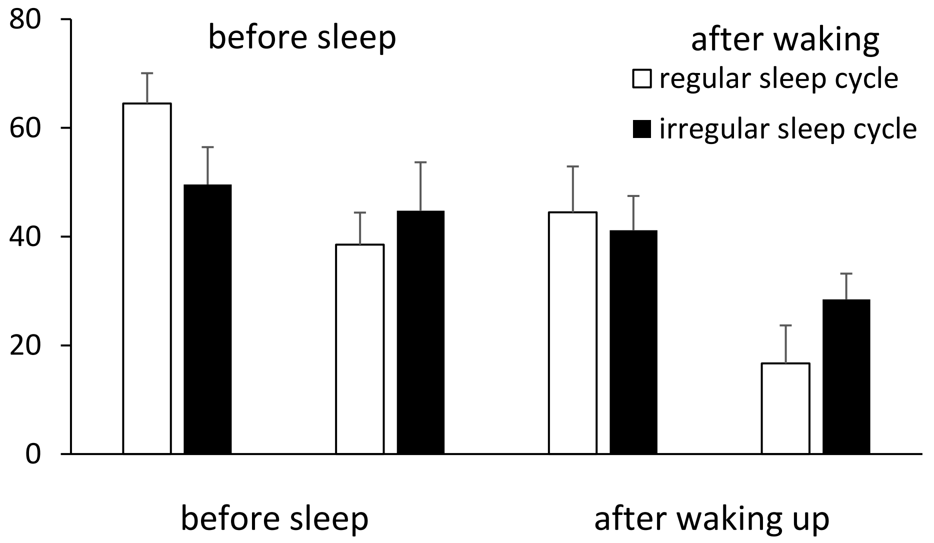

Supplemental Figure S3. %Lag0 and sleep cycles

Appendix 3 (only for reviewers): Coefficients of variation (CV) in physical acceleration before and after sleep in the older and non-older groups.

For unknown reasons, physical acceleration was significantly lower in older participants than that in non-older participants 4 h before sleep. However, it may be attributed to early circadian rhythm of physical activities in older participants. Significant differences were not observed between %CV of physical acceleration before and after sleep, which suggests variabilities in the daily activities between the non-older and older groups.

Supplemental Table S4

	Non-older (n = 50)		Older (n = 45)	
Before sleep (h)	PA (mG)	%CV	PA (mG)	%CV
5	33.1±4.0	143.9±11.3	41.4±4.7	110.3±6.2
4	40.1±4.9	141.5±15.4	24.9±2.7*	122.1±8.1
3	28.9±4.0	150.6±8.2	23.3±2.7	123.7±8.2
2	22.9±2.4	142.8±8.8	23.9±4.1	129.5±7.1
1	28.2±2.2	124.6±5.4	27.4±2.6	119.2±6.0
Sleep				
After waking up (h)	PA (mG)	%CV	PA (mG)	%CV
1	45.3±4.3	100.8±4.7	40.3±5.9	98.1±6.5
2	44.0±4.2	124.4±9.4	48.9±9.7	116.1±13.6
3	47.5±4.7	146.9±14.6	43.3±7.6	117.7±7.8
4	43.5±5.6	148.3±14.5	49.4±4.8	103.9±6.6
5	44.6±4.8	136.2±10.3	47.7±4.6	103.7±5.0

*p < 0.05, non-older vs. older

CV, coefficients of variation; PA, physical acceleration

Appendix 4 (only for reviewers): Circadian rhythm of %lag0

Supplemental Figure S5 depict the changes in %lag0 every hour between PA and HFnu. %Lag0 between PA and HFnu before sleep was slightly higher than that after waking up in both the older and non-older groups. It was difficult to calculate %lag0 during sleep due to lower PA.

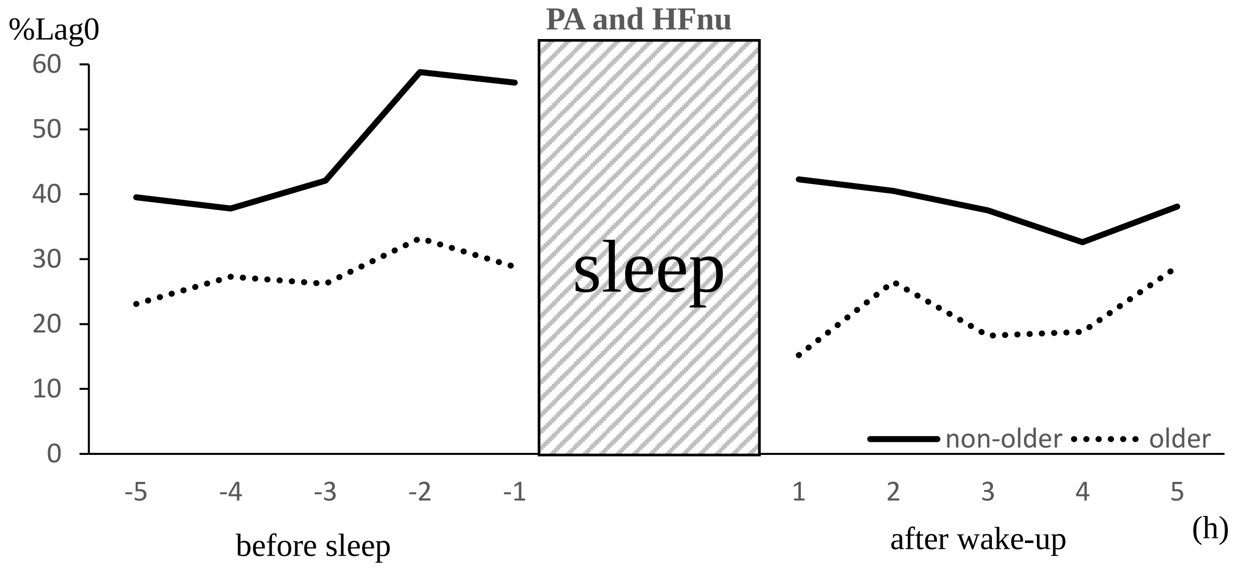

Supplemental Figure S5. %Lag0 between physical acceleration (PA) and heart rate variability index [HFnu = HF/(LF+HF)]

Appendix 5: %Lag0 between PA and HR

In our preliminary studies, we analyzed the lag between heart rate and PA. In non-older group, an hour before sleep, Cohen's D effect size of %lag0 between heart rate and PA had a medium value. %lag0 between heart rate and PA was similar to that between HFnu and PA with medium values of Cohen's D effect size in non-older group, an hour before sleep. Almost all participants in non-older and older groups indicated approximately %lag0 = 100 between PA and heart rate during one hour before sleep and after waking up. Therefore, the frequency domain method was more specific than heart rate methods to detect the coordination.

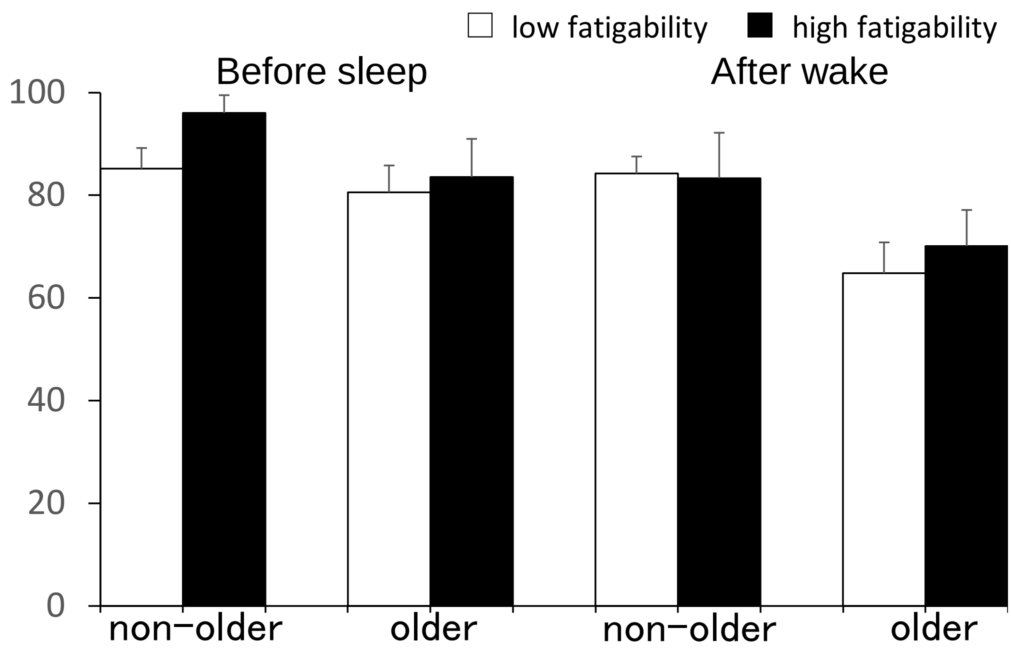

Supplemental Figure S6. %Lag0 between PA and HR

Supplemental Table S7. Comparisons of HRV and HR methods of effect size and p value

	before sleep				after wake			
	non-older		older		non-older		older	
	HRV	HR	HRV	HR	HRV	HR	HRV	HR
Cohen's D effect size	**0.67**	**0.51**	0.22	0.10	0.62	0.04	0.53	0.17
p value	**<0.05**	**>0.05**	>0.05	>0.05	>0.05	>0.05	>0.05	>0.05
